# Amyloid-beta uptake by blood monocytes is reduced with ageing and Alzheimer’s disease

**DOI:** 10.1038/s41398-020-01113-9

**Published:** 2020-12-08

**Authors:** Si-Han Chen, Ding-Yuan Tian, Ying-Ying Shen, Yuan Cheng, Dong-Yu Fan, Hao-Lun Sun, Chen-Yang He, Pu-Yang Sun, Xian-Le Bu, Fan Zeng, Juan Liu, Juan Deng, Zhi-Qiang Xu, Yang Chen, Yan-Jiang Wang

**Affiliations:** 1 Department of Neurology and Centre for Clinical Neuroscience, Daping Hospital, Third Military Medical University, Chongqing, China; 2 The Institute of Brain and Intelligence, Third Military Medical University, Chongqing, China; 3 Chongqing Key Laboratory of Ageing and Brain Diseases, Chongqing, China; 4 Department of Health Management, Daping Hospital, Third Military Medical University, Chongqing, China; 5 State Key Laboratory of Trauma, Burn and Combined Injury, Daping Hospital, Third Military Medical University, Chongqing, China; 6 Center for Excellence in Brain Science and Intelligence Technology, Chinese Academy of Sciences, Shanghai, China

**Keywords:** Diseases, Psychiatric disorders

## Abstract

Deficits in the clearance of amyloid β-protein (Aβ) play a pivotal role in the pathogenesis of sporadic Alzheimer’s disease (AD). The roles of blood monocytes in the development of AD remain unclear. In this study, we sought to investigate the alterations in the Aβ phagocytosis function of peripheral monocytes during ageing and in AD patients. A total of 104 cognitively normal participants aged 22–89 years, 24 AD patients, 25 age- and sex-matched cognitively normal (CN) subjects, 15 Parkinson’s disease patients (PD), and 15 age- and sex-matched CN subjects were recruited. The Aβ uptake by blood monocytes was measured and its alteration during ageing and in AD patients were investigated. Aβ_1-42_ uptake by monocytes decreased during ageing and further decreased in AD but not in PD patients. Aβ_1-42_ uptake by monocytes was associated with Aβ_1-42_ levels in the blood. Among the Aβ uptake-related receptors and enzymes, the expression of Toll-like receptor 2 (TLR2) was reduced in monocytes from AD patients. Our findings suggest that monocytes regulate the blood levels of Aβ and might be involved in the development of AD. The recovery of the Aβ uptake function by blood monocytes represents a potential therapeutic strategy for AD.

## Introduction

Alzheimer’s disease (AD) is the most common neurodegenerative disorder affecting 35 million elderly individuals^1^. Its mechanism remains unclear, and no disease-modifying therapies are currently available. A large amount of evidence suggests that deficit in the clearance of amyloid β-protein (Aβ), which leads to the cerebral accumulation of Aβ, plays a pivotal role in the development of sporadic AD^2^.

Recent studies show that a series of AD risk gene mutations are associated with immune responses and endocytosis, and these include ATP-binding cassette transporter A7, CD33, triggering receptor expressed on myeloid cells-2 (TREM2) and complement receptor 1. These findings suggest that dysfunction of innate immunity, mainly involving microglia and peripheral myeloid cells, is a critical reason for AD^3–7^. Indeed, studies have suggested that the reduced Aβ uptake capacity of microglia in the brain is a major mechanism underlying the development of AD^8,9^. However, the alterations in the functions and roles of peripheral myeloid cells during ageing and in AD remains unclear.

While resident microglia play a key role in the clearance of Aβ in the brain, approximately 40–60% of Aβ generated in the brain is estimated to diffuse into the blood and be cleared in the periphery, implying that the peripheral system also plays an essential role in clearing Aβ from the brain^10–12^. It remains undetermined how this brain-derived Aβ is cleared in the periphery. Blood monocytes are the counterparts of microglia in the periphery. Some studies have demonstrated that monocytes are more effective at neuroprotection, neuroinflammation regulation and Aβ clearance than microglia in AD^13–17^. Therefore, monocytes might play a critical role in the clearance of brain-derived Aβ in the periphery.

In the present study, we aimed to investigate alterations in Aβ uptake by peripheral monocytes during ageing in cognitively normal (CN) subjects and in sporadic AD patients and to evaluate the role of peripheral monocytes in the uptake of Aβ in the blood.

## Methods

### Study subjects

A total of 104 CN participants aged 22–89 years, 24 AD patients, 25 age- and sex-matched CN subjects, 15 Parkinson’s disease (PD) patients and 15 age- and sex-matched CN subjects were recruited from Daping Hospital between March 2017 and May 2019. Another 25 CN subjects were enrolled for plasma collection. Based on our preliminary data, a minimum sample size of 13 patients and 13 CNs were estimated to be necessary to show a difference in Aβ uptake abilities by monocytes between AD patients and CNs with a power of 80% and an *α* of 5%.

Subjects were not eligible if they had a family history of dementia; had a concomitant neurologic disorder except for AD and PD; were in a state of obvious infection or inflammation potentially affecting the status of blood cells; had severe cardiac, pulmonary, hepatic, renal diseases or any kinds of tumour; had any potent haematopathy, including acute monocytic leukaemia and myelodysplastic syndrome, during the recovery period of agranulocytosis; had autoimmune diseases, including rheumatoid arthritis and systemic lupus erythematosus; had an endocrine system disease, including Cushing syndrome and thyroid disorders; and declined to participate in the study.

The study was approved by the ethics committee of Daping Hospital. Written consent was obtained from participants or their legal representatives.

### Clinical assessment

The clinical evaluation was performed by following the protocol described in our previous studies^18^. In brief, demographic data including age, sex, education level and occupation were collected on admission. The medical history, including current medications, prior head trauma and surgery, prior gas poisoning, schizophrenia, hypothyroidism, coronary heart diseases, atrial fibrillation, cerebrovascular diseases, chronic obstructive pulmonary disease, chronic hepatitis, chronic renal insufficiency, hypertension, diabetes mellitus, hypercholesterolaemia and regular use of non-steroidal anti-inflammatory or prescription drugs, was collected from the medical records and a formal questionnaire.

Cognitive status was assessed using a neuropsychological battery that included Minimum Mental State Examination (MMSE), Activities of Daily Living and Montreal Cognitive Assessment (MoCA). Subjects with abnormal performance in MMSE or MoCA assessment were further subjected to neuropsychological tests, including Clinical Dementia Rating, Pfeiffer Outpatient Disability Questionnaire and Hachinski Ischaemic Score (for assessing significant vascular diseases). Subjects with abnormal cognition were further subjected to a brain computed tomography/magnetic resonance imaging investigation and blood tests for thyroxine, vitamin B12, folic acid and HIV/syphilis to rule out metabolic and infectious reasons for cognitive decline.

Dementia was diagnosed based on Diagnostic and Statistical Manual of Mental Disorders, Fourth Edition criteria. The diagnosis of probable AD was made according to the criteria of the National Institute of Neurological and Communicative Diseases and Stroke and the Alzheimer Disease and Related Disorders Association. Idiopathic PD was diagnosed according to the Parkinson’s Disease Society Brain Bank criteria^19^.

### Blood sampling

To avoid possible circadian rhythm effects, the sampling conditions, including sampling timing and fasting state, were consistent among AD and PD patients and their matched CN subjects. A portion of fasting blood samples was aliquoted for measuring complete blood cell counts, fasting glucose, thyroxin, creatinine, urea, uric acid, aspartate aminotransferase, alanine aminotransferase and total cholesterol levels. For another portion of blood, plasma was separated within 30 min after sampling and stored at −80 °C for further analysis of Aβ. For Aβ uptake-related assay, the blood samples were applied for the isolation of peripheral blood mononuclear cells (PBMCs) within 2 h after blood draw.

### Isolation of blood monocytes

Fresh heparinized blood was diluted with phosphate-buffered saline (PBS; 1:1 ratio; vol/vol). PBMCs were isolated by density gradient centrifugation using Ficoll-Hypaque (TBD Science, Tianjin, China), and mononuclear sections were collected and washed with PBS three times. A portion of the PBMCs was used for the Aβ uptake assay, and the other portion of PBMCs was used for monocyte isolation by CD14 Microbeads (Miltenyi Biotec, Bergisch Gladbach, Germany) and passed through a magnetic activated cell sorting (MACS) column for the positive selection of CD14^+^ cells, according to the manufacturer’s instructions. The remaining PBMCs were frozen at a concentration of 1–2 × 10^6^ cells/ml in 10% dimethyl sulfoxide (Sigma-Aldrich, Saint Louis, USA)/90% foetal calf serum (vol/vol Gibco, CA, USA) for future use.

### Aβ uptake assay

**Fig. 1:**
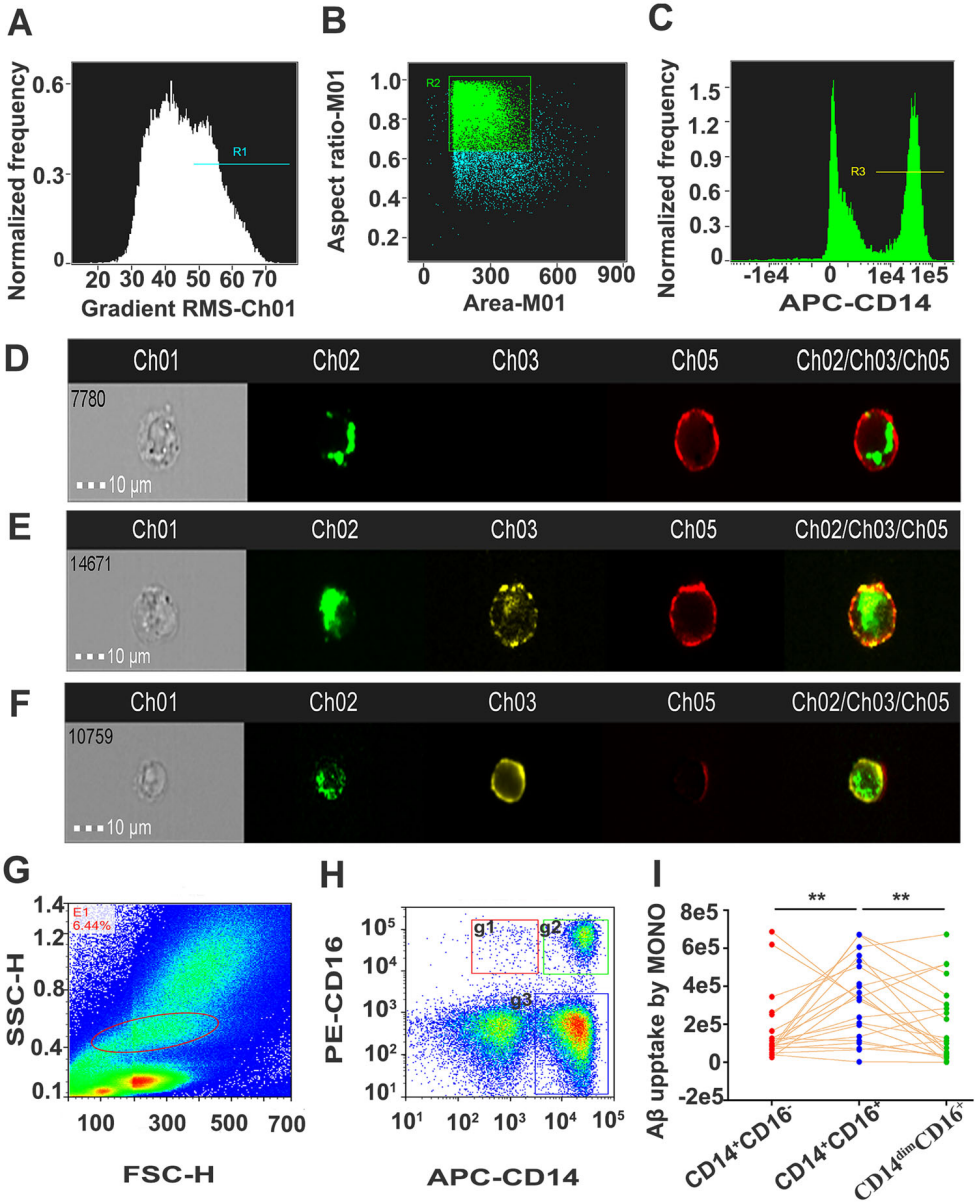
Imaging flow cytometry of Aβ_1-42_ uptake of monocyte subsets. **A** Single cells were selected from debris by gating on cells in focus, **B** followed by gating on the area and aspect ratio of the brightfield image. **C** Cells with high-intensity labelling of CD14 were chosen as monocytes. Monocytes were stained with APC-conjugated anti-CD14 mAb (red) and PE-conjugated anti-CD16 mAb (yellow), whereas FITC-conjugated Aβ_1-42_ is shown in green. Images of FITC-labelled Aβ_1-42_ uptake by the classic CD14^+^CD16^−^ monocyte subset (**D**), intermediate CD14^+^CD16^+^ monocyte subset (**E**) and non-classical CD14^dim^CD16^+^ monocyte subset were collected (**F**). **G**, **H** For the measurement of Aβ_1-42_ uptake by monocyte subsets, monocytes were gated based on FSC-H and SSC-H. Three monocyte subsets, including classical monocyte subset CD14^+^CD16^−^ (**g1**), intermediate monocyte subset CD14^+^CD16^+^ (**g2**) and non-classical monocyte subset CD14^dim^CD16^+^ (**g3**), were gated based on the expression of CD14 and CD16. **I** *n* = 44 per group, a paired *t* test, two sided. **p < 0.01. MONO monocytes, FSC-H forward scatter height, SSC-H side scatter height, Aβ amyloid-β protein.

Isolated PBMCs were resuspended in RPMI medium with 10% foetal calf serum and 1% penicillin/streptomycin and adjusted to a concentration of 2 × 10^6^ cells/ml. To test the uptake of Aβ, PBMCs were incubated with FITC-Aβ_1-42_ (2 μg/ml) (GL Biochem, Shanghai, China) overnight at 37 °C in a 5% CO_2_ incubator. Following incubation, the cell suspensions were discarded, and adherent cells were detached from the well plate by 0.25% trypsin and washed with fluorescence-activated cell sorting (FACS) buffer twice. Then the cell suspensions were preincubated with Human TruStain FcX (Biolegend, CA, USA) on ice for 20 min to avoid producing a high background by the non-specific binding of the Fc receptor expressed on immune cells to the Fc fragment of the fluorophore-labelled antibody. Then the cell suspensions were washed and stained with the following fluorophore-labelled antibodies according to the corresponding manufacturer’s instructions (BD, NJ, USA): allophycocyanin (APC)-anti human CD14, phycoerythrin (PE)-anti human CD16, APC-mouse IgG2a, κ isotype control, PE-mouse IgG2a, and κ isotype control. Following incubation, the cells were washed twice with FACS buffer and fixed with 1% paraformaldehyde. Flow cytometry was performed on a NovoCyte Flow Cytometer (ACEA Biosciences, CA, USA) after appropriate compensation. Monocytes were gated using forward and side scatter, and monocyte subsets were identified by differential expression of CD14 and CD16 (Fig. 1G, H). The data were analysed by the NovoExpress software based on forward and side scatter and the mean fluorescence intensity. To maintain consistent testing conditions, a gating strategy was designed and applied equivalently across all study samples.

### Imaging flow cytometry (IFC)

IFC was performed according to a previous report^20^. In brief, the procedures for labelling surface markers were the same as those used for conventional flow cytometry, which was described above. IFC was performed on a two-camera ISX with the INSPIRE acquisition software (Amnis, NJ, USA). Excitation lasers used for analysis included 5 mW 405 nm, 100 mW 488 nm and 150 mW 642 nm. A 2.5 mW 785 nm laser was used for internal calibration to provide a scatter signal and measure speed beads. FITC and PE were excited by the 488 nm laser, and the emission was captured in the ranges of 505–560 nm (Ch02) and 560–595 nm (Ch03). APC was excited by the 647 nm laser, and the emission was captured in the wavelength range of 642–745 nm (Ch05). In total, 25,000 events were acquired, and all images were captured with the ×20 objective and a cell classifier (threshold) applied to the bright field channel (Ch01) to exclude small particles (Fig. 1A, B). Monocytes were identified using the Amnis IDEAS software. Cells with high-intensity labelling of the CD14 marker were chosen as monocytes (Fig. 1C—R3). For analysis of Aβ_1-42_ internalization feature, high-intensity labelling of FITC-conjugated Aβ_1-42_ monocytes were chosen, following selection of single cells with high-intensity labelling of APC-conjugated CD14 (Supplemental Fig. 1a–c). The internalization of FITC-Aβ_1-42_ by monocytes is defined as the ratio of the intensity inside the cell to the intensity of the entire cell. Internalized cells typically have positive scores while cells with little internalization have negative scores. Cells with scores around 0 have a mix of internalization and membrane intensity. Followed by gating on internalized monocytes with positive score and negative score, composite of images of channel 2 (FITC-Aβ_1-42_) and channel 5 (APC-CD14) are shown (Supplemental Fig. 11d, e).

### Confocal microscopy

Human monocytes were enriched by plastic adhesion overnight and seeded onto a collagen-coated MatTek culture dish with 10 µm cover slide on the bottom. For FITC-Aβ_1-42_ uptake, monocytes were incubated with FITC-Aβ_1-42_ (2 μg/ml) overnight at 37 °C in a 5% CO_2_ incubator. Cells were washed three times with ice-cold PBS and stained with anti-human CD14 monoclonal antibody (Abcam, Cambridge, Britain) at 4 °C. Cells were washed with PBS and stained with Alexa Fluor 594 Anti-Mouse IgG (Invitrogen, CA, USA) for 1 h and then mounted with Mounting Medium with 4′,6-diamidino-2-phenylindole (Santa Cruz, CA, USA). The dishes were examined by OLYMPUS confocal microscope.

### Aβ_1-42_ assay

Plasma Aβ_1-42_ levels were measured using an ultra-sensitive single molecule array (SIMOA) on the Simoa HD-1 Analyser (Quanterix, Lexington, MA), as previously described^21^. SIMOA technique implies immunocapture of the target protein on magnetic beads, which are trapped in femtoliter volume wells, followed by the addition of enzyme-labelled detection antibody and accurate digital quantification. The high analytical sensitivity of this technique allows for pre-dilution of plasma samples, thus contributing to reducing matrix interferences. It has been widely used and been validated useful in numerous studies^22^.

### Measurement of Aβ uptake-related receptors in monocytes

The staining of Aβ uptake-related receptors, including Toll-like receptor 2 (TLR2), TREM2, CD36, CD33 and macrophage scavenger receptor 1 (SCARA1), was performed by flow cytometry. Following CD14-positive selection by MACS, 1.0 × 10^5^ monocytes were preincubated with Human TruStain FcX (Biolegend, CA, USA) on ice for 20 min. For cell surface staining, cells were incubated with monoclonal antibodies against APC-anti human CD33, BB515-anti human CD282, Percp-CyTM5.5-anti human CD36, BV421-anti human MSR1 (Biolegend, CA, USA) and PE-anti human TREM2 for 15 min, washed via centrifugation twice and fixed with 1% paraformaldehyde. Cells were acquired on a FACS Navios (Beckman, CA, USA), and analyses were performed using the FlowJo v10 software.

### Measurement of Aβ-degrading enzymes in monocytes

Western blotting was performed as previously described. PBMCs were lysed in RIPA buffer. Samples were subjected to electrophoresis on sodium dodecyl sulfate–polyacrylamide gel electrophoresis (8–12% acrylamide) gels. The blots were probed with antibodies against cathepsin D (1:1000, monoclonal, Arigobio), cathepsin S (1:1000, monoclonal, Arigobio) and β-actin (1:1000, monoclonal, Sigma-Aldrich). The protein bands were scanned using the Odyssey scanner software (Li-COR Bioscience, CA, USA) and quantified by Quantity One 6.0. The band density was normalized to that of β-actin in the same sample.

### Statistical analysis

For each statistical analysis, appropriate tests were selected based on whether or not the data were normally distributed (D’Agostino–Pearson normality test). Differences in demographic characteristics were assessed by Chi-square test. For normally distributed data, statistical comparisons between two groups were made using Student’s *t* test or paired *t* test (or Student’s *t* test with Welch’s correction if the *F*-test showed significantly different variances between groups). For non-normally distributed data, Wilcoxon matched-pairs test or Mann–Whitney test was used, where appropriate. Specifically, values of monocyte Aβ uptake of AD, PD and their age- and sex-matched CN were logarithmically transformed to an approximate normal distribution. Student’s *t* test was used to determine the differences between groups. A Spearman correlation or covariate correlation analysis was utilized to analyse the association of Aβ_1-42_ uptake with ageing or Aβ_1-42_, Aβ_1-40_ levels, and Aβ_1-42_/Aβ_1-40_ ratio in the plasma, respectively. The trajectory of Aβ_1-42_ uptake with age was modelled using Fit spline/Lowess (cubic) spline. All statistical analyses were performed with the GraphPad Prism v5.0 software. The data are expressed as the mean ± SEM, unless otherwise stated. *P* values < 0.05 (two sided) were considered significant.

## Results

### Aβ_1-42_ uptake by monocyte subsets

In humans, peripheral monocytes are divided into three subsets based on the expression of the cell-surface markers CD14 (a pattern recognition receptor for lipopolysaccharide) and CD16 (Fcγ III receptor), including non-classic monocytes with low CD14 expression and high CD16 expression (CD14^dim^CD16^+^ or CD14^−^CD16^+^), intermediate monocytes with high or intermediate CD14 and CD16 expression (CD14^+^CD16^+^), and classic monocytes with high CD14 expression but very low or negative CD16 expression (CD14^+^CD16^−^)^23^. We found that all subsets of monocytes could take up Aβ_1-42_ (Fig. 1D–F). Intracellular location of Aβ_1-42_ was further validated by confocal stacks (Supplemental Fig. 1). For IFC, the median internalization erode of FITC-Aβ_1-42_ by monocyte was 1.598, and the positive rate of Aβ_1-42_ internalized monocytes almost reached 93.1%, indicating that Aβ proteins were taken up by monocytes (Supplemental Figs. 1 and 2). The CD14^+^CD16^+^ subset had the highest uptake of Aβ_1-42_ among the three subsets, with no significant difference in Aβ_1-42_ uptake between the CD14^+^CD16^+^ and CD14^dim^CD16^+^ subsets (Fig. 1I).

### Aβ_1-42_ uptake by monocyte subsets during ageing

**Fig. 2:**
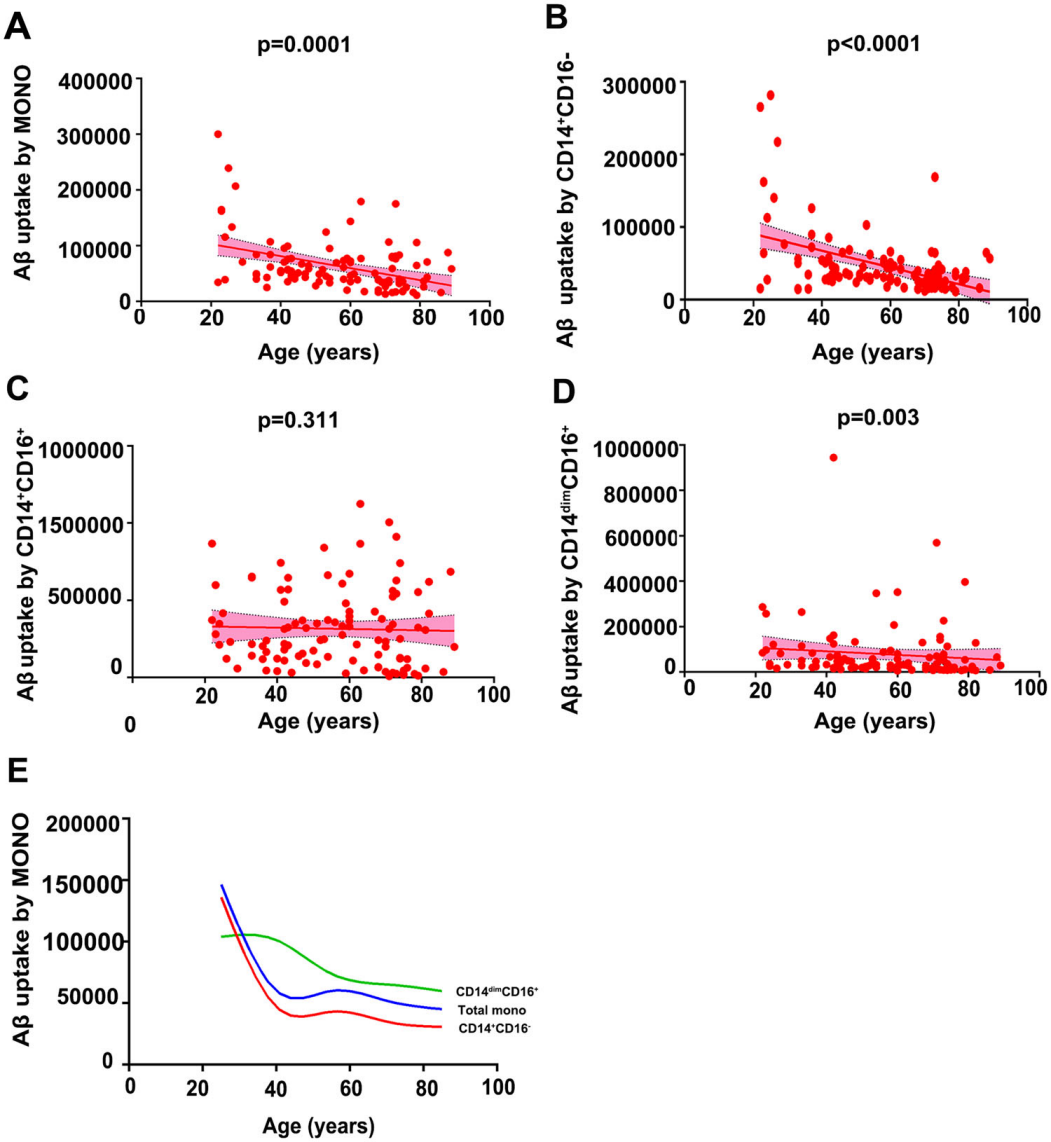
Correlation and trajectory of Aβ_1-42_ uptake by monocyte subsets relative to age. Spearman correlation analysis was utilized to investigate the correlation between age and the uptake of Aβ_1-42_ by all monocytes (**A**), by the CD14^+^CD16^−^ subset (**B**), by the CD14^+^CD16^+^ subset (**C**) and by the CD14^dim^CD16^−^ subset (**D**). **E** The trajectory of Aβ_1-42_ uptake by total monocytes, CD14^dim^CD16^+^ and the CD14^+^CD16^−^ subsets relative to age was modelled using Fit spline/Lowess (cubic) spline. *n* = 104. MONO monocytes, Aβ amyloid-β protein.

Then we measured Aβ_1-42_ uptake by monocytes in 104 CN subjects aged 22–89 years who were not different in sex among the different age groups (Supplemental Table 1). Aβ_1-42_ uptake by the total monocyte population was correlated with age; that is, the older the age was, the lower the Aβ_1-42_ uptake level (*γ* = −0.3661, *P* = 0.0001). Among the three subsets, Aβ_1-42_ uptake was correlated with age in the CD14^+^CD16^−^ (*γ* = −0.374, *P* < 0.0001) and CD14^dim^CD16^+^ subsets (*γ* = −0.285, *P* = 0.003), but not in the CD14^+^CD16^+^ subset (*γ* = −0.100, *P* = 0.311; Fig. 2A–D). Aβ_1-42_ uptake by total monocytes and the CD14^+^CD16^−^ subset decreased rapidly in the group aged 20–40 years, but the reduction rate became relatively slow after 40 years of age. Aβ_1-42_ uptake by CD14^dim^CD16^+^ subset decreased rapidly in the group aged 40–60 years, but the reduction rate became relatively slow after 60 years of age. This result suggests that the decrease in Aβ uptake is a life-long process that may be different among the three monocyte subsets and occurs prior to the cerebral deposition of Aβ (Fig. 2E).

### Correlation between Aβ_1-42_ uptake by monocytes and Aβ levels in the blood

**Fig. 3:**
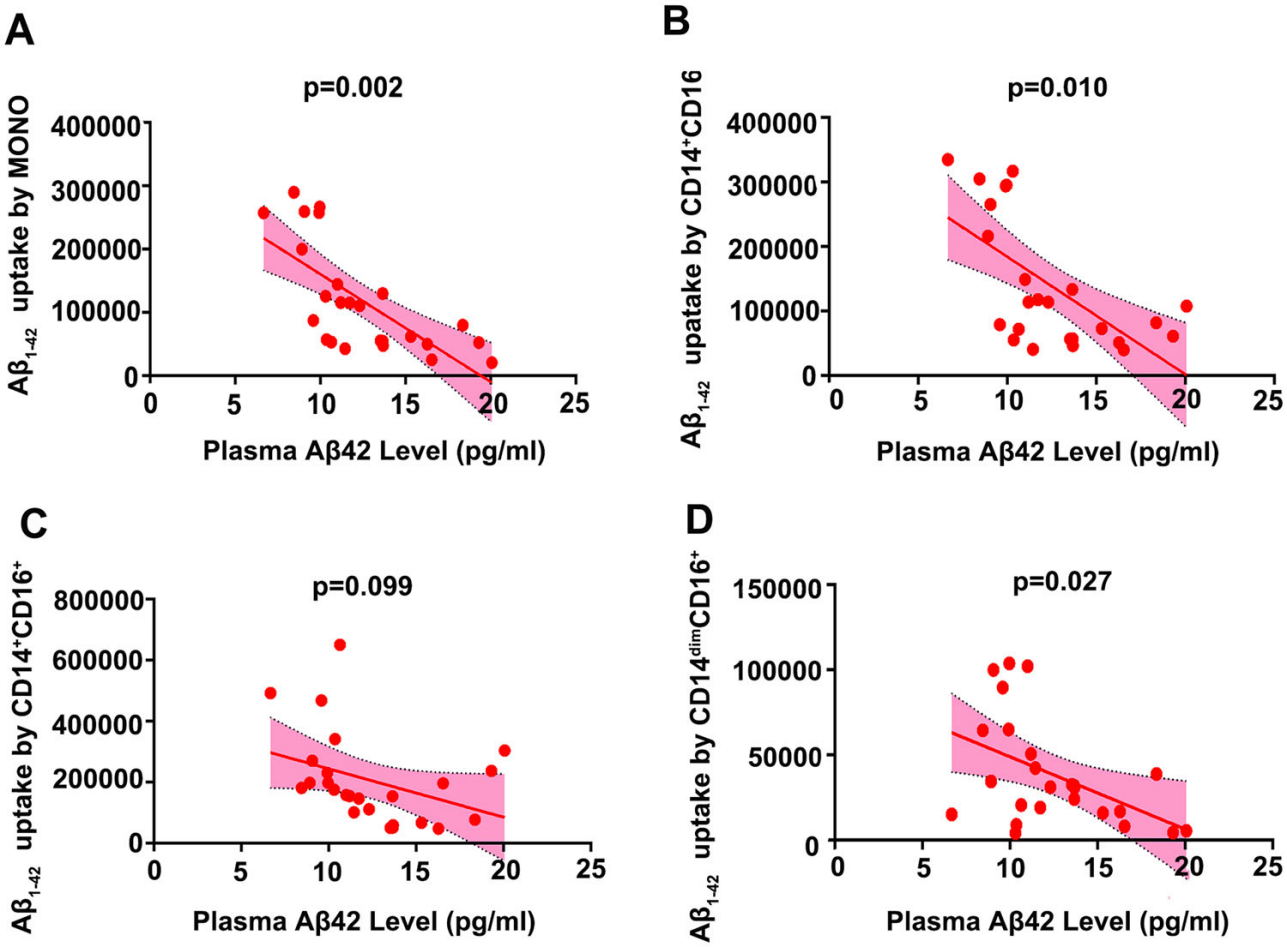
Association of monocyte Aβ_1-42_ uptake with plasma Aβ_1-42_ levels in CN subjects. Correlations between plasma Aβ_1-42_ and the uptake of Aβ_1-42_ by all monocytes (**A**), by the CD14^+^CD16^−^ subset (**B**), by the CD14^+^CD16^+^ subset (**C**) and by the CD14^dim^CD16^−^ subset (**D**). *n* = 25, covariate correlation analysis. MONO monocytes, Aβ amyloid-β protein.

**Fig. 4:**
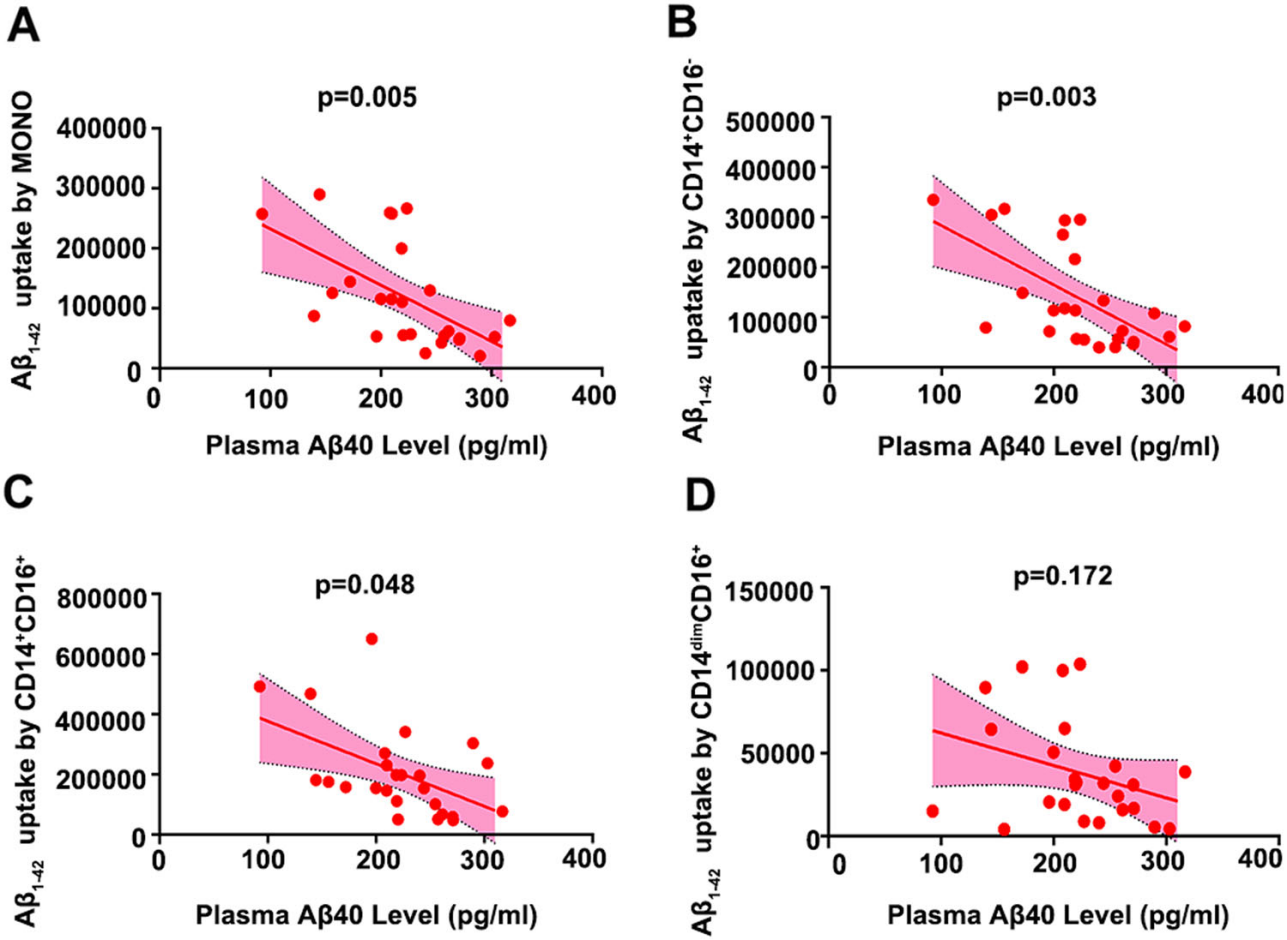
Association of monocyte Aβ_1-42_ uptake with plasma Aβ_1-40_ levels in CN subjects. Correlations between plasma Aβ_1-42_ and the uptake of Aβ_1-42_ by all monocytes (**A**), by the CD14^+^CD16^−^ subset (**B**), by the CD14^+^CD16^+^ subset (**C**) and by the CD14^dim^CD16^−^ subset (**D**). *n* = 25, covariate correlation analysis. MONO monocytes, Aβ amyloid-β protein.

After controlling the confounding factors of age, sex, vascular risk factors and *APOE* ε4 genotype, the partial correlation analysis showed that Aβ_1-42_ uptake by total monocytes was significantly correlated with plasma Aβ_1-42_ levels (*γ* = −0.668, *P* = 0.002) in CN subjects (*n* = 25, mean age 60.54 ± 17.21 years). Among them, Aβ_1-42_ uptake by both CD14^+^CD16^−^ (*γ* = −0.576, *P* = 0.010) and CD14^dim^CD16^+^ (*γ* = −0.506, *P* = 0.027) subsets, but not the CD14^+^CD16^+^ subset (*γ* = −0.390, *P* = 0.099), was correlated with plasma Aβ_1-42_ levels (Fig. 3). Moreover, Aβ_1-42_ uptake by total monocytes was significantly correlated with plasma Aβ_1-40_ levels (*γ* = −0.615, *P* = 0.005; Fig. 4A). Among them, Aβ_1-42_ uptake by CD14^+^CD16^−^ (*γ* = −0.640, *P* = 0.003) and CD14^+^CD16^+^ (*γ* = −0.458, *P* = 0.048) subsets, but not the CD14^dim^CD16^+^ (*γ* = −0.327, *P* = 0.172) subset, was correlated with plasma Aβ_1-40_ levels (Fig. 4B–D). Aβ_1-42_ uptake by CD14^dim^CD16^+^ (*γ* = −0.470, *P* = 0.042) subset, but not the total monocyte (*γ* = −0.235, *P* = 0.334), CD14^+^CD16^−^ (*γ* = −0.039, *P* = 0.874) and CD14^+^CD16^+^ (*γ* = −0.080, *P* = 0.746) subsets, was correlated with plasma Aβ_1-42_/Aβ_1-40_ ratio (Supplemental Fig. 3).

### Aβ_1-42_ uptake by monocytes from AD and PD patients and CN subjects

**Fig. 5:**
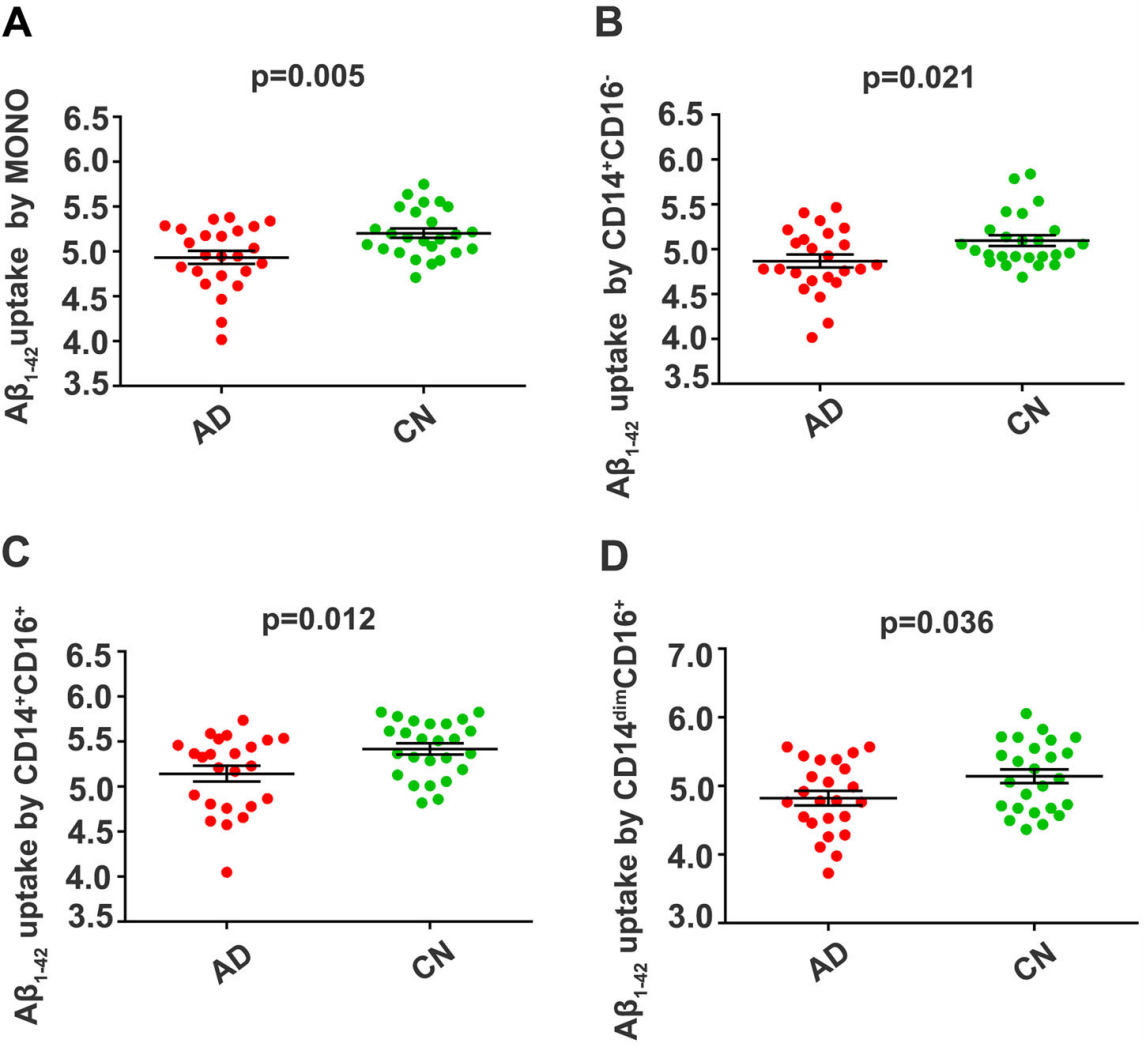
Comparison of Aβ_1-42_ uptake by monocyte subsets between AD patients and CN subjects. Compared with CN controls, AD patients had decreased Aβ_1-42_ uptake by total monocytes (**A**), the CD14^+^CD16^−^ subset (**B**), the CD14^+^CD16^+^ subset (**C**) and the CD14^dim^CD16^−^ subset (**D**). *n* = 24 for AD; *n* = 25 for CN, mean ± SEM, Student’s *t* test, two sided. AD Alzheimer’s disease, CN cognitively normal control, MONO monocytes, Aβ amyloid-β protein.

To investigate whether the alteration in Aβ_1-42_ uptake by monocytes is specific to AD patients, 24 AD patients, 15 age- and sex-matched PD patients and their matched CNs were enrolled (Supplemental Tables 2 and 3). There were no significant differences in sex, age, years of education, *APOE* ε4 genotype, comorbidities, including hypertension, hyperlipidaemia and diabetes mellitus, or medications between the matched groups. We found that Aβ_1-42_ uptake by total monocytes and the various subsets was lower in AD patients than in CNs (Fig. 5). There were no significant differences in Aβ_1-42_ uptake by total monocytes and their subsets between PD patients and CNs (Supplemental Fig. 4). These results suggest that Aβ_1-42_ uptake by monocytes might be specifically decreased in AD patients.

### Expression of Aβ_1-42_ uptake-related receptors and Aβ-degrading enzymes in monocytes of AD patients

**Fig. 6:**
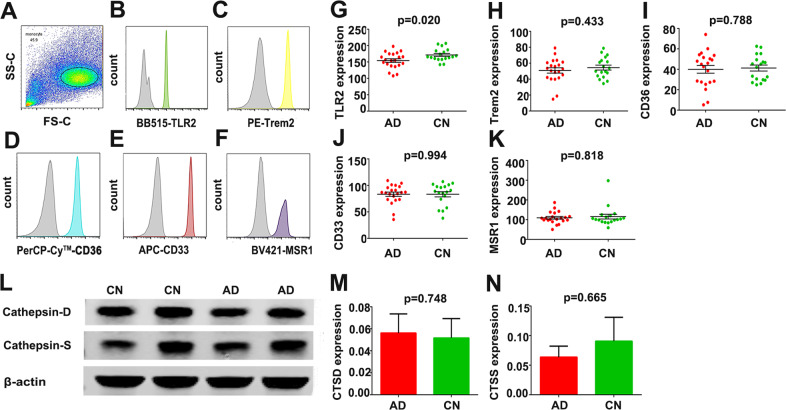
Detection of Aβ_1-42_ uptake-related receptors and Aβ-degrading enzymes in the monocytes of AD patients. **A** Following CD14-positive selection by magnetic activated cell sorting, monocytes were identified by flow cytometry by their forward-side-scatter appearance (*n* = 21 for AD; *n* = 18 for CN, mean ± SEM). **B**–**F** The expression of TLR2, TREM2, CD36, CD33 and MSR1 was assessed in monocytes, and the expression levels are depicted in representative histograms; grey curves indicate negative control staining (*n* = 21 for AD; *n* = 18 for CN, mean ± SEM; Student’s *t* test, two sided). **G** The expression level of TLR2 was decreased in AD patients compared with CN subjects (*n* = 21 for AD; *n* = 18 for CN, mean ± SEM; Student’s *t* test, two sided). **H**–**K** There was no significant difference between AD patients and CN controls in the expression levels of TREM2, CD36, CD33 and MSR1 (*n* = 21 for AD; *n* = 18 for CN, mean ± SEM; Mann–Whitney test or Student’s *t* test, two sided). **I**–**M** There was no significant difference between AD patients and CN controls in the expression levels of Aβ-degrading enzymes, including cathepsin D and cathepsin S (*n* = 12 for AD; *n* = 12 for CN, mean ± SEM; Mann–Whitney test or Student’s *t* test, two sided). AD Alzheimer’s disease, CN cognitively normal control, Aβ amyloid β-protein, TLR2 Toll-like receptor 2, TREM2 triggering receptor expressed on myeloid cells 2, SCARA1 macrophage scavenger receptor 1.

In AD, we tested five major Aβ-uptake receptors involved with myeloid cell-mediated physiological uptake of Aβ, including TLR2, TREM2, CD36, CD33 and SCARA1. The expression of TLR2 was lower in AD patients than in CNs. However, no differences were observed in the expression of receptors, including TREM2, CD36, CD33 and SCARA1, between AD patients and CNs (Fig. 6A–K). To investigate whether internalized Aβ_1-42_ was effectively digested by monocytes in AD, we tested the expression of cathepsin D and cathepsin S, two main lysosomal aspartic and cysteine proteases, which were suggested to mediate Aβ_1-42_ degradation^24–26^. There were no significant differences in the protein levels of Aβ-degrading enzymes, including cathepsin D and cathepsin S, between AD patients and CNs (Fig. 6L–M).

## Discussion

Increasing evidence suggests that failure of Aβ clearance leads to accumulation of Aβ plaques over 20–30 years prior to the onset of dementia^27,28^. A previous study has demonstrated ineffective uptake of Aβ in AD patients. However, whether there exist specific alterations of Aβ uptake in different monocyte subsets and the role of peripheral monocytes in Aβ uptake in AD remains unclear. Ageing is an important factor for the development of AD^29^. We found that Aβ_1-42_ uptake by monocytes decreased as age increased. Consistently, a previous study showed that the internalization of Aβ_1-42_ by aged human blood cell-derived monocytes was lower than that by human umbilical cord blood cell-derived monocytes^30^. But it did not show the trajectories of Aβ uptake by different monocyte subsets during ageing. In our study, it is worth noting that the decrease in Aβ uptake by classic monocyte subset and non-classic subset began at the age of 20 years and 40 years respectively, suggesting that the decrease in Aβ uptake ability is a life-long process that may be different among monocyte subsets and occurs prior to brain Aβ deposition. Despite the impact of ageing, the Aβ uptake ability of monocytes is further decreased in AD patients, implying that compromised Aβ uptake by monocytes is involved in AD pathogenesis^31–33^.

In humans, peripheral monocytes can be divided into non-classic, intermediate and classic monocyte subsets^23^. These monocyte subsets may have different functions in AD. As reflected by our findings, the intermediate subset had the highest Aβ uptake ability, while there was no significant difference in Aβ uptake between the classic and non-classic subsets in CN controls. The Aβ uptake ability was mainly decreased in the classic and non-classic subsets during ageing, except for the most effective intermediate subset. However, Aβ uptake ability was decreased in all subsets in AD patients. A previous study reports that CD14^+^CD16^+^ subset is the main producer of interleukin (IL)-10 upon stimulation of TLRs^34^. However, dysfunctional CD14^+^CD16^+^ subset not only affect Aβ clearance in AD but also may lead to reduction of IL-10, which is an anti-inflammatory cytokine and protective factor for neurogenesis and cognitive preservation, thus exacerbating the pathogenesis of AD^14^. These results suggest that there is an overall decrease in Aβ uptake by all three monocyte subsets in AD patients, implying that the mechanisms underlying the alteration in Aβ uptake ability by monocytes in AD patients are different from those associated with ageing.

The mechanisms underlying the decreased Aβ uptake ability by monocytes during ageing and AD remain to be investigated. We found that the decrease of Aβ uptake could be partially due to deficits in Aβ recognition by monocytes, as reflected by the reduced expression of TRL2 in monocytes. TRL2 belongs to a type I transmembrane pattern recognition receptor and acts as a natural innate immune receptor for Aβ uptake through the formation of a receptor complex with CD14. Deletion or inhibition of the CD14-TLR receptor complex will impair fibrillary Aβ_1-42_ uptake in human monocytes and delay cognitive decline in a mouse model of AD^35,36^. Previous studies have explored the intracellular processing and degradation of Aβ within monocyte. After receptor-mediated Aβ endocytosis, early endosomes are formed and further combined with lysosomes for degradation^37^. Thereinto, cathepsin D and cathepsin S, which are the two main lysosomal aspartic and cysteine proteases, were demonstrated to mediate Aβ degradation. However, we did not find any differences in the expression of these two Aβ-degrading enzymes between AD patients and CN controls, suggesting that dysfunctional Aβ recognition could be particularly important for the decrease in Aβ uptake by monocytes in AD patients.

The decrease in Aβ uptake by monocytes seems specific to AD, as it was not changed in PD patients compared with CN controls in our study. Additional evidence indicates that the Aβ uptake ability is correlated with blood Aβ levels in CN subjects, that is, the greater the Aβ uptake ability is, the lower the blood Aβ levels. These findings suggest that monocytes might play a critical role in clearing Aβ from the blood.

In conclusion, our findings are of significance to the understanding of the pathogenesis of sporadic AD. Aβ in the brain can be transported to the peripheral blood^38–40^, and the clearance of Aβ in the periphery has been suggested to substantially contribute to the clearance of Aβ from the brain^10,41^. Therefore, the decrease in Aβ uptake by monocytes could play a significant role in the development of sporadic AD. The recovery of the Aβ uptake function of blood monocytes may represent a therapeutic strategy for AD. The potential methods include adoptive transfer of healthy monocytes and bone marrow transplantation, modification of monocyte with Aβ-degrading enzymes and activation of monocytes by cytokines or agents^42^.

## Supplementary information

Supplemental material
