# Supplementary material for: Amyloid-beta uptake by blood monocytes is reduced with ageing and Alzheimer’s disease
Source: Transl Psychiatry. 2020 Dec 8;10:423. doi: 10.1038/s41398-020-01113-9 (PMC7722845; doi:10.1038/s41398-020-01113-9)
Supplement: Supplementary file 1 — Supplemental material [file 41398_2020_1113_MOESM1_ESM.docx]

**SUPPLEMENTAL INFORMATION**

**Supplemental figures**

**Supplemental figure 1.**

**
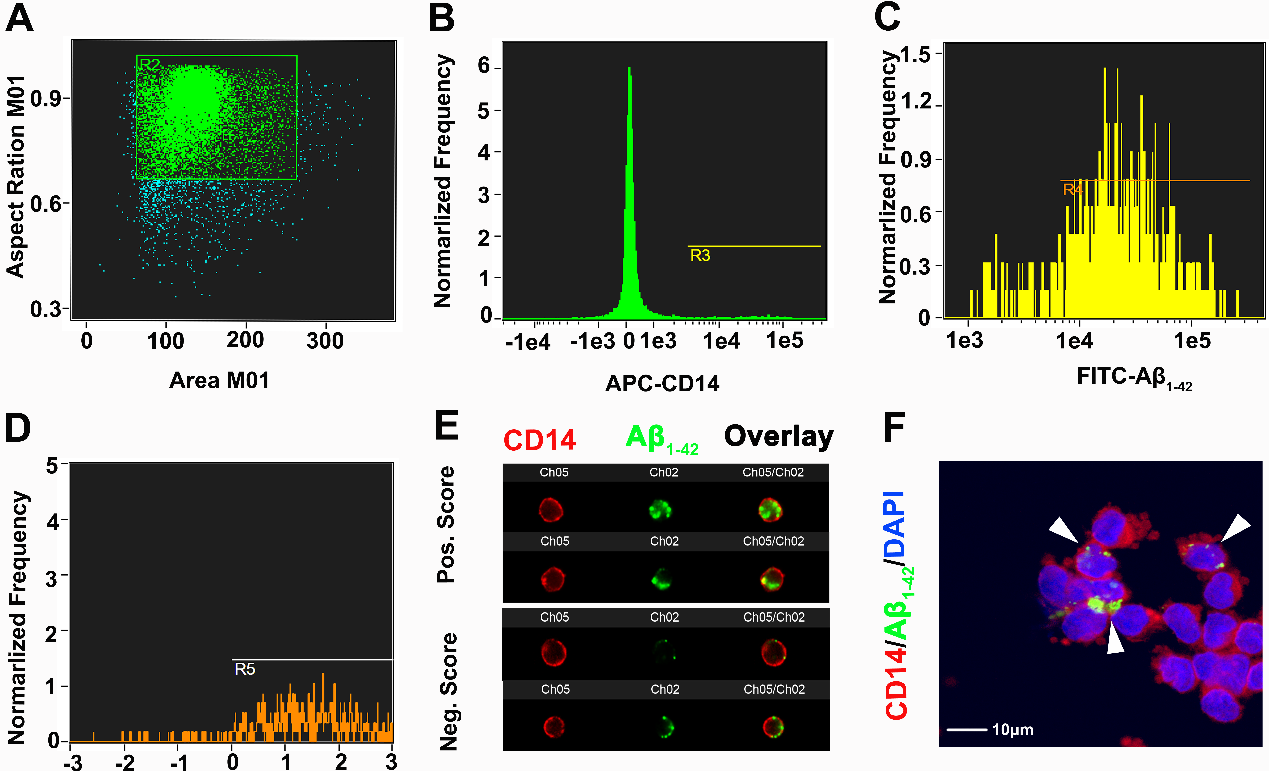
**

**Supplemental figure 1. Imaging flow cytometry of Aβ_1-42_ internalization feature. (a)** Selection of single and circular cells using the aspect ratio vs area plots of bright field channel (R2). **(b)** Cells with high-intensity labelling of APC conjugated CD14 were chosen as monocytes (R3). **(c)** Aβ_1-42_ positive monocytes were selected by high-intensity labelling of FITC conjugated Aβ_1-42_ (R4). **(d)** The internalization feature, defined as the ratio of the intensity inside the cell to the intensity of the entire cell, showed a clear separation between clusters of negative internalization and positive internalization (R5). **(e)** Followed by gating on internalized monocytes with positive score and negative score, composite of images of channel 2 (FITC- Aβ_1-42_) and channel 5 (APC-CD14) were shown. **(f)** Confocal stack of images of Aβ_1-42_ uptake by human monocytes. Monocytes were stained with Alexa594 conjugated anti-CD14 monoclonal antibody (red) and counter stained with DAPI (blue), while FITC conjugated Aβ_1-42_ were shown in green. MONO, monocytes; Aβ, amyloid-β protein, Pos, positive; Neg, negative.

**Supplemental figure 2.**

**
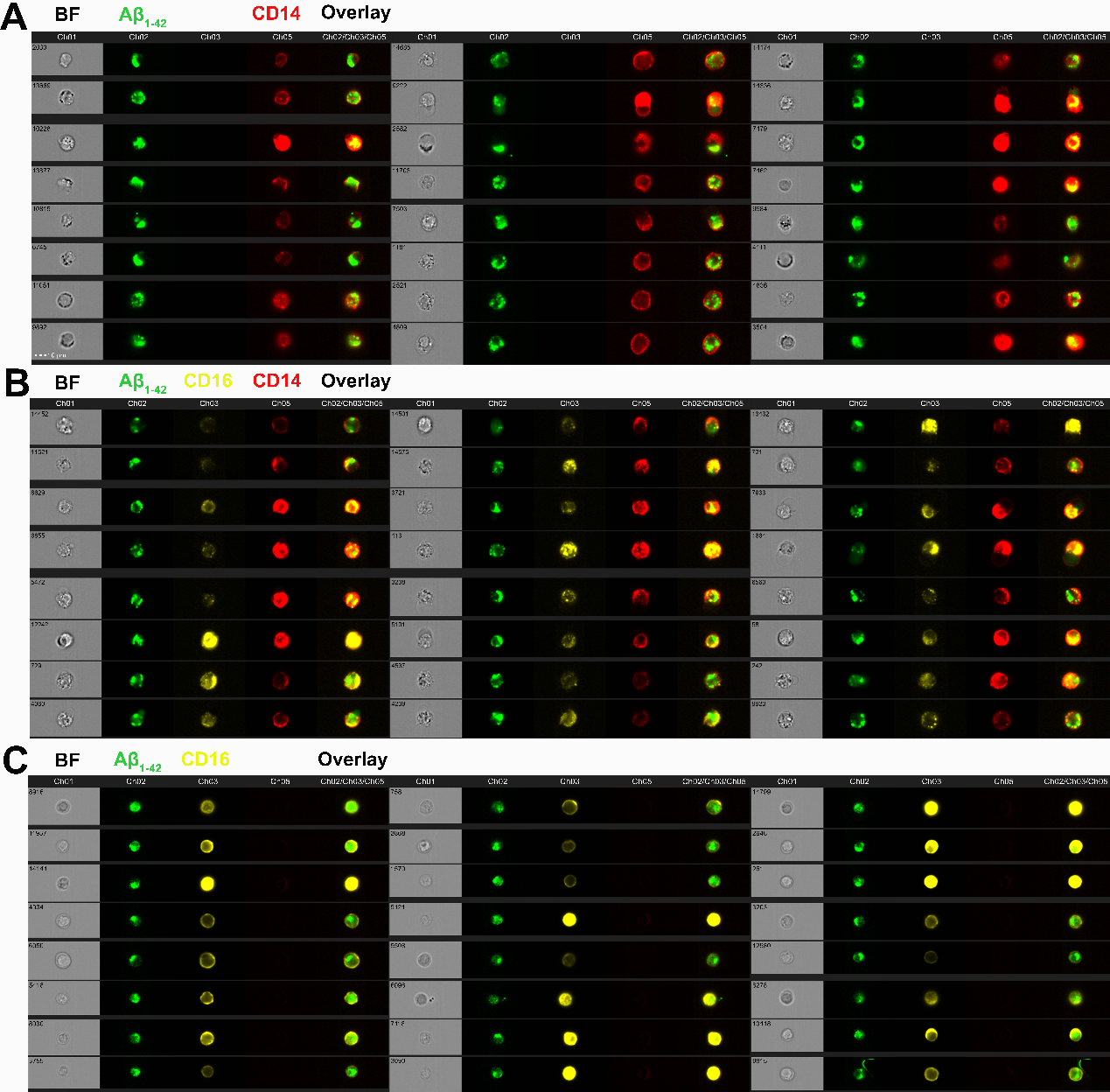
**

**Supplemental figure 2. Imaging flow cytometry of intracellular Aβ_1-42_ uptake by monocyte subsets.** Monocytes were stained with APC-conjugated anti-CD14 mAb (red) and PE-conjugated anti-CD16 mAb (yellow), and FITC-conjugated Aβ_1-42_ is shown in green. Overlay images of FITC-labelled Aβ_1-42_ intracellular uptake by the classical CD14^+^CD16^-^ monocyte subset **(a)**, intermediate CD14^+^CD16^+^ monocyte subset **(b)** and the non-classical CD14^dim^CD16^+^ monocyte subset **(c)** were collected. BF, bright field.

**Supplemental figure 3.**


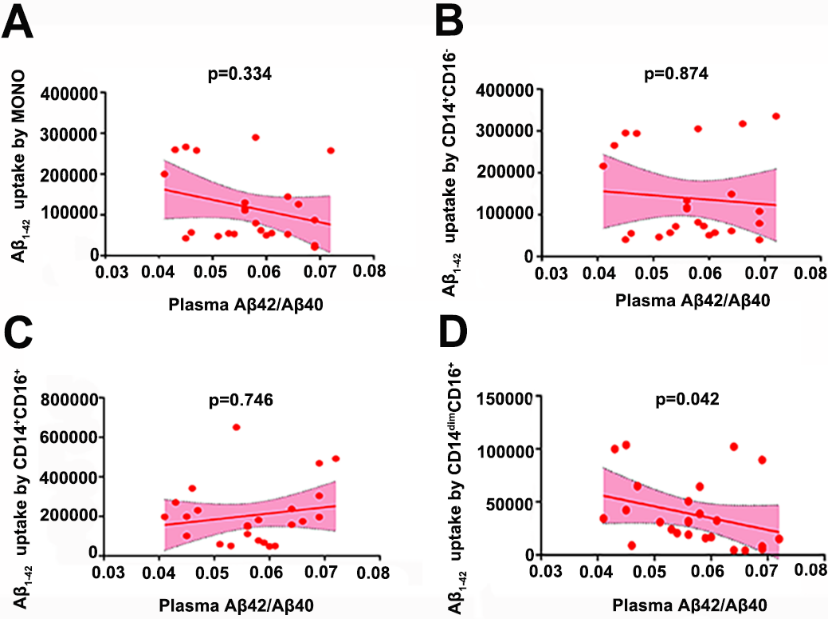


**Supplemental figure 3. Association of Aβ_1-42_ uptake by monocytes with plasma Aβ_1-42_/Aβ_1-40_ level.** Correlations between plasma Aβ_1-42_/ Aβ_1-40_ and uptake of Aβ_1-42_ by all monocytes **(a)**, by the CD14^+^CD16^-^ subset **(b)**, by the CD14^+^CD16^+^ subset **(c)**, and by the CD14^dim^CD16^+^ subset **(d)**. N=25, covariate correlation analysis. MONO, monocytes; Aβ*,* amyloid β-protein.

**Supplemental figure 4.**

**
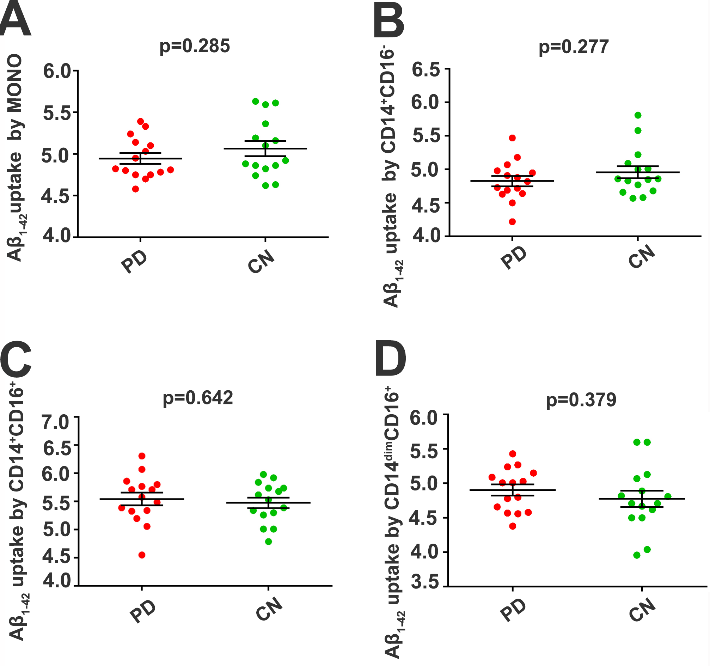
**

**Supplemental figure 4.** **Comparison of Aβ_1-42_ uptake by monocyte subsets between PD patients and CN subjects**. **a-d** There were no significant differences between PD patients and the age- and sex-matched CN controls in Aβ_1-42_ uptake by total monocytes, the CD14^+^CD16^-^ subset, the CD14^+^CD16^+^ subset and the CD14^dim^CD16^-^ subset. N=15 per group, mean±SEM, Student’s *t* test, two-sided. PD, Parkinson’s disease; CN, cognitively normal control; MONO, monocytes; Aβ, amyloid-β protein.

**Supplemental table 1. Characteristics of cognitively normal subjects**

| **Age range** | **<= 30 years**  **(n=10)** | **31-45 years**  **(n=25)** | **46-60 years**  **(n=25)** | **61-75 years**  **(n=32)** | **> 75 year**  **(n=12)** | **P value** |
| --- | --- | --- | --- | --- | --- | --- |
| **Age, y [mean (SD)]** | 25.0 (2.9) | 39.8 (4.0) | 57.2 (3.0) | 69.6 (4.0) | 83.0 (5.0) | <0.0001 |
| **Female, n (%)** | 5 (50.0) | 13 (52.0) | 12 (48.0) | 15 (46.9) | 5 (41.7) | 0.98 |
| **Comorbidities** |  |  |  |  |  |  |
| **Hypertension, n (%)** | 0 (0.0) | 2 (8.0) | 2 (8.0) | 4 (12.5) | 2 (16.7) | 0.70 |
| **Diabetes mellitus, n (%)** | 0 (0.0) | 1 (4.0) | 1 (4.0) | 2 (6.3) | 1 (8.3) | 0.90 |
| **Hyperlipidemia, n (%)** | 1 (10.0) | 2 (8.0) | 2 (8.0) | 2 (6.25) | 1 (8.3) | 1.00 |

**Supplemental Table 2. Demographic data of AD patients and age- and sex-matched CN controls**

|  | **AD**  **(n=24)** | **CN**  **(n=25)** | **P value** |
| --- | --- | --- | --- |
| **Age, y [mean (SD)]** | 70.2 (7.6) | 69.8 (8.2) | 0.87 |
| **Male, n (%)** | 12 (50.0) | 13 (52.0) | 1.00 |
| **Years of education, mean (SD)** | 9. 4 (3.6) | 9.4 (12.0) | 0.70 |
| ***APOE* ε4 carriers, n (%)** | 7 (29.2) | 3 (8.0) | 0.17 |
| **MMSE, mean (SD)** | 10.2 (8.7) | 28.0 (1.1) | <0.0001 |
| **CDR, mean (SD)** | 2.1 (0.9) | 0.0 | <0.0001 |
| **ADL, mean (SD)** | 47.5 (18.9) | 20.8 (1.0) | <0.0001 |
| **Comorbidities** |  |  |  |
| **Hypertension, n (%)** | 6 (25.0) | 4 (16.0) | 0.50 |
| **Diabetes mellitus, n (%)** | 1 (4.2) | 3 (12) | 0.61 |
| **Hyperlipidemia, n (%)** | 2 (8.3) | 1 (4.0) | 0.61 |
| **Medication, n (%)** |  |  |  |
| **Hypertension** | 5 (20.8) | 3 (12.0) | 0.46 |
| **Diabetes mellitus** | 1 (4.2) | 3 (12.0) | 0.61 |
| **Hyperlipidemia** | 1 (4.2) | 1 (4.0) | 1.00 |

AD, Alzheimer’s disease; CN, cognitively normal control; SD, standard deviation; MMSE, Mental State Examination; ADL, Activities of Daily Living; CDR, Clinical Dementia Rating.

**Supplemental Table 3. Demographic data of PD patients and age- and sex-matched CN controls**

|  | **PD**  **(n= 15)** | **CN**  **(n= 15)** | **P values** |
| --- | --- | --- | --- |
| **Age, y [mean (SD)]** | 65.8 (10.8) | 67.3 (10.0) | 0.70 |
| **Male, n (%)** | 8 (53.3) | 8 (53.3) | 1.00 |
| **Years of education, mean (SD)** | 9.2 (2.1) | 9.1 (2.7) | 0.88 |
| ***APOE* ε4 carriers, n (%)** | 2 (13.3) | 1 (6.7) | 1.00 |
| **Comorbidities** |  |  |  |
| **Hypertension, n (%)** | 0 (0.0) | 1 (6.7) | 1.00 |
| **Diabetes mellitus, n (%)** | 0 (0.0) | 1 (6.7) | 1.00 |
| **Hyperlipidemia, n (%)** | 1 (6.7) | 0 (0.0) | 1.00 |
| **Medication, n (%)** |  |  |  |
| **Hypertension** | 0 (0.0) | 1 (5.0) | 1.00 |
| **Diabetes mellitus** | 0 (0.0) | 1 (5.0) | 1.00 |
| **Hyperlipidemia** | 0 (0.0) | 0 (0.0) | / |

PD, Parkinson’s disease; CN, cognitively normal control; SD, standard deviation.
